# Navigating the Therapeutic Tightrope: Refractory Lithium-Induced Nephrogenic Diabetes Insipidus in a Patient With Advanced Chronic Kidney Disease

**DOI:** 10.7759/cureus.110503

**Published:** 2026-06-09

**Authors:** Bhupender Kumar, Munir Babar, Mishal Nisar

**Affiliations:** 1 Diabetes and Endocrinology, Worcestershire Acute Hospitals NHS Trust, Worcester, GBR

**Keywords:** diabetes insipidus, hypernatraemia, hypernatraemia polyuria diabetes insipidus, lithium-induced diabetes insipidus, nephrogenic diabetes insipidus, polydipsia-polyuria syndrome

## Abstract

Lithium is the most common and effective mood stabiliser in the management of bipolar disorder. Lithium-induced nephrogenic diabetes insipidus (NDI) is a well-recognised complication of long-term mood stabiliser therapy. However, management becomes exponentially more complex when therapeutic options, such as thiazides or amiloride, are contraindicated due to advanced chronic kidney disease (CKD) or electrolyte instability. We are reporting the case of a man in his 60s with a 40-year history of lithium use who presented with acute-on-chronic kidney injury (AKI) and severe hypernatraemia (164 mmol/L) refractory to standard desmopressin therapy. This case highlights the limitations of conventional NDI treatments in the setting of a low estimated glomerular filtration rate (eGFR) and the necessity of intensive, multidisciplinary care.

## Introduction

Antidiuretic hormone (ADH) is a peptide hormone which is released from the posterior pituitary. It is also known as arginine vasopressin (AVP) and is essential to maintain the body's water homeostasis and serum osmolality. ADH augments water reabsorption in the renal collecting ducts by regulating the expression of aquaporin-2 (AQP2) on the type 2 arginine vasopressin receptor (AVPR2) [1-3]. Nephrogenic diabetes insipidus (NDI) has been classified as congenital and acquired. It is characterised by polyuria and decreased urine osmolality despite normal or elevated ADH secretion [2,3]. Various causes of acquired NDI have been described including renal failure, electrolyte disturbances, and medications including lithium [3]. Lithium remains a cornerstone for bipolar and schizoaffective disorders, but long-term exposure often results in NDI by inhibiting adenylate cyclase and downregulating AQP2 expression [3,4]. NDI has been the most common adverse effect reported in patients on long-term lithium therapy [3,5]. In many patients, this impairment persists even after lithium cessation due to irreversible structural remodelling of the collecting ducts [6]. Lithium is widely used for the treatment of bipolar and schizoaffective disorders as one of the most effective mood stabilisers. However, prolonged use reduces the responsiveness of the renal collecting ducts to ADH through multiple mechanisms, leading to loss of ability to conserve water and concentrate urine, resulting in polyuria [7]. When NDI is compounded by chronic kidney disease (CKD), the standard management algorithm, which often includes thiazide diuretics or amiloride, is frequently precluded by the risk of worsening acute-on-chronic kidney injury (AKI) or hyperkalaemia. We report here a case of NDI who presented with increased confusion. Despite being on desmopressin for five years, the patient's sodium was high, and he still had the symptoms of NDI.

## Case presentation

A man in his 60s with a significant psychiatric and medical history presented from a care facility following an acute decline in mental and functional status. His history included schizophrenia, bipolar affective disorder, type 2 diabetes mellitus, and lithium-induced stage 3b CKD in 2018, and his baseline estimated glomerular filtration rate (eGFR) was 36. His extensive medication list included lithium, sodium valproate, risperidone, and levothyroxine. Upon admission, the patient was hemodynamically stable but exhibited signs of severe dehydration and reduced consciousness (Glasgow Coma Scale (GCS) score: 13/15). His AKI was precipitated by reduced intake and dehydration. Initial laboratory investigations (Table 1) revealed a sodium level of 155 mmol/L (rising to 164 mmol/L) during the first 24 hours, creatinine of 338 µmol/L, baseline eGFR of 15 mL/min/1.73 m^2^, potassium of 5.8 mmol/L, and lithium level of 1.0 mmol/L (mildly elevated). He was a diagnosed patient of NDI, and during this admission, his serum osmolality was 366 mOsm/kg, his urine osmolality was 294 mOsm/kg, and his urine output ranged from 2500 mL to 3950 mL in 24 hours despite being on desmopressin 200 mcg three times a day. His brain MRI revealed age-related involutional and mild small vessel white matter ischaemic changes. The clinical course was complicated by refractory hypernatraemia despite initial fluid resuscitation. The patient's condition deteriorated because of worsening GCS due to persistent hypernatraemia and encephalopathy requiring ICU admission for ventilatory support and precise electrolyte titration.

**Table 1:** Key laboratory findings on presentation Na: sodium; K: potassium; eGFR: estimated glomerular filtration rate; CRP: C-reactive protein; Hb: haemoglobin; WBC: white blood cell; Plt: platelets; ECG: electrocardiogram; AKI: acute-on-chronic kidney injury; CKD: chronic kidney disease

Investigation	Result	Reference range	Comment
Na	155 mmol/L	135-145 mmol/L	Elevated (hypernatraemia)
K	5.8 mmol/L	3.5-5.0 mmol/L	Elevated (hyperkalaemia)
Urea	17.4 mmol/L	2.5-7.8 mmol/L	Elevated
Creatinine	338 µmol/L	62-106 µmol/L	Elevated (AKI on CKD)
eGFR	15 mL/min/1.73 m^2^	>90 mL/min/1.73 m^2^	Severely reduced
Lithium level	1.0 mmol/L	0.6-0.8 mmol/L	Mildly elevated
CRP	7 mg/L	<1 mg/L	Mildly elevated
Hb	103 g/L	130-170 g/L	Mild anaemia
WBC count	8.8×10⁹/L	4.0-11.0×10⁹/L	Within normal range
Plt	123×10⁹/L	150-450×10⁹/L	Mild thrombocytopenia
Calcium	2.3 mmol/L	2.2-2.6 mmol/L	No abnormalities detected
Phosphate	0.86 mmol/L	0.8-1.5 mmol/L	No abnormalities detected
Head CT	No acute changes	-	Chronic small vessel disease, cerebral atrophy
ECG	Not specified	-	Advised for monitoring due to hyperkalaemia

Treatment and outcome

The patient exhibited (sub)maximal resistance to desmopressin (1-deamino-8-D-arginine vasopressin (DDAVP)), confirming the nephrogenic nature of the diabetes insipidus which has been a diagnosed clinical situation at hand. Standard NDI treatments were evaluated but deemed inappropriate, as with an eGFR of 15, the delivery of solute to the distal tubule was insufficient for thiazides to be effective and amiloride was contraindicated due to the patient's baseline hyperkalaemia and advanced CKD. His sodium level rose to 164 on the second day, and the free water deficit was calculated to be 7.4 L. Therefore, management shifted to the administration of intravenous 5% dextrose and free water via nasogastric tube according to the calculated water deficit on a daily basis alongside the immediate discontinuation of lithium (Table 2). This whole process of management was under endocrinology consultation and ICU-level monitoring. His sodium levels were gradually corrected. He was successfully extubated and stepped down to the medical ward for rehabilitative care. For the management of his bipolar disorder, his lithium was stopped, and he was switched to carbamazepine 200 mg twice a day for considering his best interest by the psychiatry department. He was discharged after a hospital stay of 26 days with a sodium level of 139, creatinine of 177, and eGFR of 33 mL/min/1.73 m^2^.

**Table 2:** Water deficit replacement followed on a daily basis NG: nasogastric

Day	Sodium	Management line
1	155	IV fluids, continued desmopressin and lithium
2	154	IV fluids, continued desmopressin and lithium
3	164	Lithium and desmopressin stopped, fluid replacement as per water deficit (IV dextrose 5% at the rate of 200 mL/hour and NG tube free water of 2.2 L/24 hours)
4	161	Fluid replacement as per water deficit (IV dextrose 5% at the rate of 160 mL/hour and NG tube free water of 2 L/24 hours)
5	159	Fluid replacement as per water deficit (IV dextrose 5% at the rate of 150 mL/hour and NG tube free water of 1.8 L/24 hours)
6	151	Fluid replacement as per water deficit (IV dextrose 5% at the rate of 130 mL/hour and NG tube free water of 1.5 L/24 hours)
7	149	Fluid replacement as per water deficit (IV dextrose 5% at the rate of 130 mL/hour and NG tube free water of 1.5 L/24 hours)
8	141	Fluid replacement to match the urine output

## Discussion

This case illustrates that NDI with AKI or CKD or both may pose difficulty in management. Every NDI patient may not respond to desmopressin, and if the patient has AKI or CKD, then nonsteroidal anti-inflammatory drugs (NSAID) and thiazide may not be advisable. Therefore, it becomes a clinical problem to manage these patients. This case serves as a stark reminder of the long-term complication of lithium treatment, a clinical phenotype where the very drug that stabilised a patient's psychiatric health for decades eventually compromises their physiological stability by disrupting the ability to respond to ADH. The pathophysiology of lithium-induced NDI is a multifactorial process involving the dysregulation of AQP2 and direct toxicity to the tubular epithelium, leading to a profound loss of urinary concentrating ability. The most commonly observed adverse effect of long‐term lithium therapy is NDI, leading to the excretion of copious amounts of dilute urine and hypernatraemia [7]. Bedford et al. described the ability of amiloride to increase the urine osmolality, and it has been shown to improve the responsiveness to AVP [8]. However, the academic challenge highlighted here is the management paradox created by comorbid renal failure. Thiazide diuretics are traditionally used to treat NDI by inducing mild volume contraction, which enhances proximal tubule reabsorption [9,10]. However, with an eGFR of 15 mL/min/1.73 m^2^, the filtered load of sodium is too low for distal-acting thiazides to exert a therapeutic effect. Amiloride is the gold standard for preventing lithium entry into the collecting ducts [7]. In this patient, however, its use was strictly precluded by pre-existing hyperkalaemia and the risk of life-threatening cardiac arrhythmias in the setting of AKI. The patient's failure to respond to desmopressin, even at high doses, underscores the "fixed" nature of NDI once chronic structural damage and fibrosis have occurred in the renal medulla. Therefore, because of this state of resistance to ADH, NDI is more difficult to treat. However, some patients may benefit from desmopressin [10]. Whenever the patient's intake is reduced for any reason, they may present with severe hypernatraemia. Therefore, early recognition of hypo-osmolar polyuria may play a crucial role in management. Urinary concentrating ability may improve to some extent if we recognise early and discontinue the lithium therapy. We treated the hypernatraemia by replacing the calculated free water deficit with IV fluids and free water given through the nasogastric tube. After the initial stabilisation of sodium, the next step was to prevent subsequent hypernatraemia for which his lithium was switched to carbamazepine along with desmopressin.

## Conclusions

The resolution of this case required shifting away from pharmacological "quick fixes" to the meticulous, ICU-guided titration of free water and the definitive cessation of the offending agent. This patient's journey from acute deterioration to successful extubation highlights a critical evolution in how we must view long-term lithium therapy in an aging population. Early vigilance is better than a late rescue. Polyuria in a lithium patient is not a benign side effect; it is a clinical marker of early collecting duct dysfunction. Physicians must proactively weigh the risks of irreversible NDI against the benefits of mood stabilisation long before the eGFR begins its terminal decline.

A multidisciplinary approach is advised for the management of such cases. When NDI is complicated by AKI or advanced CKD, management should immediately involve a team of endocrinology, nephrology, and psychiatry. Standard algorithms for hypernatraemia correction fail when the kidneys cannot conserve water. In some of the cases, the permanent structural damage can be prevented by early recognition and the discontinuation of lithium. As lithium remains a "gold standard" for mood disorders, this case challenges the academic community to refine monitoring protocols. We must transition from merely checking "therapeutic levels" to actively monitoring the "functional renal reserve" to prevent patients from reaching a management dead-end where all standard therapeutic options are lost. The combination of a possible switch from lithium and controlled replacement of water deficit should be the initial management, and the subsequent options of desmopressin and amiloride can be explored. 
